# Associations among apolipoproteins, oxidized high-density lipoprotein and cardiovascular events in patients on hemodialysis

**DOI:** 10.1371/journal.pone.0177980

**Published:** 2017-05-18

**Authors:** Hirokazu Honda, Tsutomu Hirano, Masashi Ueda, Shiho Kojima, Shinichi Mashiba, Yasuyuki Hayase, Tetsuo Michihata, Kanji Shishido, Keiko Takahashi, Nozomu Hosaka, Misa Ikeda, Daisuke Sanada, Takanori Shibata

**Affiliations:** 1 Division of Nephrology, Department of Medicine, Showa University Koto Toyosu Hospital, Tokyo, Japan; 2 Division of Nephrology, Department of Medicine, Showa University School of Medicine, Tokyo, Japan; 3 Division of Diabetes, Metabolism, and Endocrinology, Department of Medicine, Showa University School of Medicine, Tokyo, Japan; 4 Ikagaku, Research Development, Kyoto, Japan; 5 Department of Dialysis, Ebara Clinic, Tokyo, Japan; 6 Department of Dialysis, Kawasaki Clinic, Kawasaki, Japan; 7 Division of Dialysis, Kitami Higashiyama Clinic, Tokyo, Japan; The University of Tokyo, JAPAN

## Abstract

Apolipoproteins are associated with survival among patients on hemodialysis (HD), but these associations might be influenced by dysfunctional (oxidized) high-density lipoprotein (HDL). We assessed associations among apolipoproteins and oxidized HDL, mortality and cardiovascular disease (CVD) events in patients on HD. This prospective observational study examined 412 patients on prevalent HD. Blood samples were obtained before dialysis at baseline to measure lipids, apolipoproteins, oxidized LDL, oxidized HDL, high-sensitivity C-reactive protein (hs-CRP) and interleukin (IL)-6 at baseline, and HDL-C and hs-CRP were measured 12 months later. Patients were then prospectively followed-up (mean, 40 months) and all-cause mortality and composite CVD events were analyzed. Associations between variables at baseline and clinical outcome were assessed by Cox proportional hazards modeling (n = 412) and Cox hazards modeling with a time-varying covariate with HDL-C and hs-CRP (n = 369). Quartiles of apolipoproteins and oxidized HDL were not associated with all-cause mortality. However, Cox proportional hazards models with quartiles of each variable adjusted for confounders and hs-CRP or IL-6 identified apolipoprotein (apo)B-to-apoA-I ratio (apoB/apoA-I) and oxidized HDL, but not apoA-I or apoA-II, as independent risk factors for composite CVD events. These associations were confirmed by Cox proportional hazards modeling with time-varying covariates for hs-CRP. ApoB/apoA-I was independently associated with composite CVD events in 1-standard deviation (SD) increase-of-variables models adjusted for the confounders, oxidized HDL and hs-CRP. However, these associations disappeared from the model adjusted with IL-6 instead of hs-CRP, and oxidized HDL and IL-6 were independently associated with composite CVD events. Findings resembled those from Cox proportional hazards modeling using time-varying covariates with HDL-C adjusted with IL-6. In conclusion, both oxidized HDL and apoB/apoA-I might be associated with CVD events in patients on prevalent HD, while associations of apoB/apoA-I with CVD events differed between models of apoB/apoA-I quartiles and 1-SD increases, and were influenced by IL-6.

## Introduction

High-density lipoprotein (HDL) represents a main factor in reverse cholesterol transport and offers protective effects against atherosclerosis. HDL is made up of apolipoproteins and the major components of HDL are apolipoprotein (apo)A-I and apoA-II. Both apoA-II and apoA-I exert anti-atherosclerotic effects through several mechanisms [1, 2]. An ApoA-II gene transgenic animal model shows reductions in atherosclerotic lesions with anti-inflammatory and anti-oxidative activities and a strong cholesterol efflux effect [2], but the effect of cholesterol efflux and esterification of cell-derived cholesterol by apoA-II are either not or only weakly supported by in vitro studies [3, 4]. The roles of apoA-II in the course of atherogenesis and the ability of apoA-II to predict cardiovascular events have not been established in the general population [5–7].

Several studies have analyzed associations between apolipoproteins and all-cause and cardiovascular disease (CVD)-related mortality in patients undergoing dialysis, although the associations remain controversial. A recent *post hoc* analysis of the German Diabetes Dialysis (4D) study identified a positive effect of apoA-II on all-cause mortality among patients on hemodialysis (HD), whereas HDL-cholesterol (C) and apoA-I were not associated with survival in these patients [8]. On the other hand, Sato reported that apoA-I and the apoB-to-apoA-I ratio (apoB/apoA-I) were significantly associated with all-cause and CVD-related mortality in those patients [9]. The possible benefits of apoA-I and apoA-II on survival and cardiovascular risk might thus be influenced by specific conditions such as inflammation, oxidative stress and malnutrition in patients with advanced chronic kidney disease (CKD) who undergo HD.

Antioxidant enzymes can be inactivated in the presence of systemic inflammation and oxidative stress, and HDL can become pro-inflammatory through the accumulation of oxidized lipids and proteins [10]. Under such conditions, reactive oxygen species can modify the proteins present in HDL, such as apoA-I [11]. Changes caused by such oxidation disrupt the anti-inflammatory effects of HDL, resulting in dysfunctional HDL that exerts pro-inflammatory effects [12–14]. In fact, HDL is often dysfunctional in patients with advanced CKD [12,14].

Several studies have investigated associations between dysfunctional HDL and atherosclerosis, and mortality in patients with CKD under dialysis [15, 16]. We have also shown that dysfunctional HDL, estimated as oxidized HDL, was significantly associated with CVD events in cohorts of patients on prevalent HD and pre-dialysis patients with CKD [17,18]. Our previous studies examined associations among oxidized HDL, HDL-C and LDL-C, and CVD events [17] and associations between oxidized HDL and their subfractions adjusted with apoA-I, and CVD events [18]. In our previous study for cohorts of patients on prevalent HD, oxidized HDL was identified as an independent risk factor for CVD events in models adjusted with HDL-C and inflammatory biomarkers such as high-sensitivity (hs)-C-reactive protein (CRP) and interleukin (IL)-6 [17].

In patients under HD, apoA-I catabolism is significantly increased compared with the normal population, while the rate of apoA-I production remains unchanged [19]. On the other hand, the rate of apoB catabolism is markedly decreased compared with production [19]. Moreover, inflammation appears associated with lowered apoA-I [8, 20], but not apoB, in patients on HD [20]. Although apolipoproteins, as with any proteins present in HDL, would possibly be modified by systemic inflammation and oxidative stress, the metabolism of apoA-I and apoA-II may be more influenced by those conditions than the metabolism of apoB in patients under HD. Compared with apoA-I or apoA-II alone, apoB/apoA-I may thus represent a significant risk factor for all-cause death or cardiovascular events, since the predictive abilities of apoB/apoA-I for these clinical outcomes in patients under HD appear superior to those of apoA-I or apoB alone in models adjusted using CRP [9]. However, associations between oxidized HDL, IL-6, apolipoprotein-related measures such as apoA-I, apoA-II concentration and apoB/apoA-I, and CVD events have not been assessed.

Based on all these findings and issues, we hypothesized that oxidized HDL would be an independent risk factor for CVD events compared with apolipoproteins such as apoA-I or apoA-II alone or apoB/apoA-I in patients under HD. Moreover, we speculated that IL-6 would interfere with the associations among oxidized HDL, apolipoproteins and CVD events, since associations of IL-6 with CVD events may be stronger than those of CRP [21]. We therefore conducted the present study to assess associations among oxidized HDL, inflammatory biomarkers (hs-CRP and IL-6), apolipoproteins (apoA-I, apoA-II and apoB), and all-cause mortality and composite CVD events in patients undergoing HD.

## Material and methods

### Patients

The present investigation was part of an ongoing prospective cohort study of 412 outpatients, and used the same participant population described in our previous study [17]. Exclusion criteria were as described in that study: lack of blood samples; anticipated life expectancy < 6 months; or presentation with clinical signs of overt infection, acute vasculitis or liver disease [17]. Smokers were not excluded from this study. All patients provided written, informed consent to participate in the study, and all protocols were approved by the ethics committee at Showa University School of Medicine. The study was performed in accordance with the 2004 revision of the Declaration of Helsinki.

Hemodialysis protocols, including dialysis dose, were similar for all patients, as described in detail previously [17]. Patients with dyslipidemia were managed with similar medications that were prescribed according to the guidelines of the Kidney Disease Outcomes Quality Initiative (K/DOQI) [22].

### Study design

The study design was previously reported in detail [17]. Briefly, this prospective cohort study included a baseline cross-sectional analysis to assess associations between oxidized HDL, lipids, apolipoproteins and biomarkers associated with mortality and CVD events. Patients were subsequently followed-up for up to 48 months to assess the impact of oxidized HDL, lipids and apolipoproteins on the number of CVD events and mortality.

Survival analysis included time to death from all causes or from CVD-related events (mean follow-up, 40 months). We analyzed elapsed time to all causes of death or composite CVD events comprising fatal and non-fatal myocardial infarction (MI) or acute coronary syndrome (ACS), fatal cerebral infarction, or peripheral artery disease (PAD). MI or ACS was defined by clinical signs, laboratory findings and using electrocardiography and cardiac angiography, and cerebral infarction was defined from computed tomography (CT) or MRI of the brain [17]. New PAD was diagnosed from contrast-enhanced CT or peripheral angiography in patients with no previous history of PAD, or newly performed intervention therapy or amputation therapy for PAD in patients who had a history of PAD.

### Assessment of nutritional status

Nutritional status was assessed using the subjective global assessment (SGA) according to Kidney Disease Outcome Quality Initiative (K/DOQI) recommendations [23] and malnutrition was defined as a moderate to severe score on the SGA. Actual body mass index (BMI) and normalized protein catabolic rate were calculated [23].

### Biochemical methods

Non-fasting venous blood samples were collected immediately prior to the HD session at baseline, 3 days after the previous HD sessions in each patient. Routine biochemical parameters and LDL-C and HDL-C were measured using routine procedures [17]. Values of apoA-I, apoA-II and apoB, and hs-CRP were assessed using immunonephelometry. Serum oxidized LDL and IL-6 were measured using enzyme-linked immunosorbent assay (ELISA) kits, as described in detail previously [17, 18, 24]. Oxidized HDL was analyzed by ELISA using anti-oxidized monoclonal apoA-I antibody and this antibody was produced by immunization with H_2_O_2_-oxidized apoA-I, as previously described [25]. One unit per milliliter of oxidized HDL in patient sera was based on the same absorbance of 1 ng/mL of standard oxidized apoA-I as detected by ELISA. Standard oxidized apoA-I was made by H_2_O_2_ oxidation in vitro.

### Statistical analyses

Data are presented as mean ± standard deviation (SD) or median (range) unless otherwise noted, and values of *P* < 0.05 were considered indicative of statistical significance. Normally distributed variables among the three groups were compared using analysis of variance, and non-normally distributed variables were assessed using the Kruskal-Wallis test. Nominal variables among three groups were compared using the χ^2^ test. Correlations were calculated using the Spearman rank test (ρ) for non-parametric data.

Independent predictors of all-cause mortality and composite CVD events during the observation period were determined using Cox proportional hazard modeling. During follow-up, 17 patients left the study due to transfer to another hospital. These 17 patients were censored at the time of transfer to another hospital. Independent predictors of composite CVD events were also determined using Cox proportional hazard modeling with HDL-C and hs-CRP at baseline and 12 months later as time-varying covariates (n = 369). Data were analyzed using JMP Pro version 12.0.1 (SAS Institute, Cary, NC) and Stata/MP version 13.1 (StataCorp, College Station, TX).

## Results

The mean concentration of oxidized HDL was 143.3 U/mL (median, 125.0 U/mL). Positive correlations were seen between apoA-I and each of apoA-II, oxidized HDL and HDL (S1 Table). Oxidized HDL correlated inversely with apoA-I, apoA-II/apoA-I, apoB/apoA-I and hs-CRP, but showed no correlations with apoA-II, apoB or IL-6 (S1 Table).

Patient characteristics and laboratory findings according to quartiles of oxidized HDL are shown in Tables 1 and 2. Oxidized HDL quartiles were inversely associated with male gender, prevalence of diabetes mellitus, BMI, apoB/apoA-I and hs-CRP, and positively associated with hemodialysis vintage, Kt/V, HDL-C and apoA-I.

**Table 1:** Patient characteristics.

	All (n = 412)	Q1 (n = 104)	Q2 (n = 102)	Q3 (n = 104)	Q4 (n = 102)	p*
Oxidized HDL (U/mL)	125.0 (43.2, 1239.0)	81.4 (43.2, 97.0)	110.9 (97.4, 125.8)	138.9 (129.0, 162.2)	208.6 (162.6, 1239.0)	
Age (years)	63 ± 13	61 ± 13 (30, 93)	63 ± 14 (29, 89)	62 ± 13 (31, 93)	62 ± 13 (29, 90)	0.68
Gender (male, %)	62	76	67	54	50	0.002
Diabetes mellitus (%)	32	44	32	24	31	0.008
Cause of CKD (%)						0.32
Chronic glomerulonephritis	36	25	39	44	38	
Diabetic nephropathy	35	46	32	27	32	
Nephrosclerosis	14	17	15	14	12	
Polycystic kidney disease	4	2	6	5	4	
Other diseases	4	4	2	2	7	
Unknown	7	6	6	8	7	
Body mass index (kg/m^2^)	21.3 ± 3.3	22.1 ± 3.5	21.6 ± 3.1	20.8 ± 3.1	20.9 ± 3.2	0.02
History of CVD (yes, %)	47	51	49	42	46	0.48
Hemodialysis vintage (months)	105 (6, 489)	88 (6, 489)	93 (6, 424)	119 (6, 489)	156 (6, 406)	0.01
Subjective global assessment (%)	24	22	22	34	27	0.20
Kt/V	1.4 ± 0.3	1.3 ± 0.2	1.4 ± 0.3	1.4 ± 0.3	1.5 ± 0.3	0.0002
Normalized PCR (g/kg/day)	0.99 ± 0.20	0.98 ± 0.22	0.95 ± 0.19	1.01 ± 0.18	1.03 ± 0.21	0.08
Smoking habit (%)	36	41	36	33	35	0.61
ACE-I/ARB (%)	31	37	28	26	31	0.31
Statins (%)	18	21	19	14	17	0.85
All-cause mortality (%)	19	25	19	18	15	0.35
Composite CVD events (%)	27	23	21	30	40	0.01

Q1: the lowest quartile, Q2: middle-low quartile, Q3: middle-high quartile, Q4: the highest quartile.

*P value for differences of the variables among oxidized HDL quintiles. CKD: chronic kidney disease, CVD: cardiovascular disease, Normalized PCR: normalized protein catabolic rate, ACE-I/ARB: angiotensin converting enzyme inhibitor / angiotensin receptor blocker.

**Table 2:** Laboratory findings at baseline.

	All (n = 412)	Q1 (n = 104)	Q2 (n = 102)	Q3 (n = 104)	Q4 (n = 102)	P*
Oxidized HDL (U/mL)	125.0 (43.2, 1239.0)	81.4 (43.2, 97.0)	110.9 (97.4, 125.8)	138.9 (129.0, 162.2)	208.6 (162.6, 1239.0)	-
Hemoglobin (g/dL)	10.0 ± 1.0	9.9 ± 1.0	10.0 ± 1.0	9.8 ± 1.0	10.0 ± 1.0	0.37
Albumin (g/dL)	3.8 ± 0.3	3.8 ± 0.4	3.9 ± 0.3	3.8 ± 0.3	3.8 ± 0.4	0.24
Creatinine (mg/dL)	11.4 ± 2.8	11.0 ± 3.0	11.8 ± 2.9	11.5 ± 2.9	11.9 ± 2.6	0.09
Calcium (mg/dL)^†^	9.1 ± 0.7	9.1 ± 0.6	9.3 ± 0.8	9.4 ± 0.7	9.3 ± 0.7	0.01
Phosphate (mg/dL)	5.6 ± 1.2	5.5 ± 1.2	5.7 ± 1.2	5.7 ± 1.4	5.5 ± 1.3	0.45
Total-cholesterol (mg/dL)	153.2 ± 31.4	152.4 ± 29.4	151.3 ± 31.3	152.0 ± 28.0	153.9 ± 34.2	0.79
HDL-cholesterol (mg/dL)	46.9 ± 15.0	39.1 ± 11.5	47.1 ± 14.2	49.4 ± 15.0	51.9 ± 16.0	<0.0001
LDL- cholesterol (mg/dL)	80.6 ± 23.3	79.1 ± 24.1	81.7 ± 23.0	81.8 ± 22.1	76.0 ± 20.7	0.15
ApoA-I (mg/dL)	117.1 ± 24.7	107.1 ± 20.0	119.0 ± 23.7	121.3 ± 23.5	127.5 ± 28.2	<0.0001
ApoA-II/ (mg/dL)	20.2 ± 4.1	19.5 ± 3.7	20.8 ± 4.0	20.9 ± 4.1	20.4 ± 4.8	0.055
ApoA-II/apoA-I	0.17 ± 0.03	0.18 ± 0.03	0.18 ± 0.03	0.18± 0.03	0.16 ± 0.03	<0.001
ApoB (mg/dL)	66.5 ± 17.6	68.1 ± 19.4	67.6 ± 17.2	68.6 ± 18.5	64.3 ± 15.9	0.29
ApoB/apoA-I	0.59 ± 0.22	0.65 ± 0.22	0.59 ± 0.20	0.60 ± 0.26	0.53 ± 0.17	0.0003
Oxidized LDL (mU/L)	63.7 ± 32.1	66.3 ± 29.6	64.4 ± 32.9	60.0 ± 36.2	65.3 ± 29.6	0.09
Hs-CRP (mg/dL)	0.09 (0.005, 10.4)	0.16 (0.012, 7.57)	0.11 (0.013, 10.39)	0.07 (0.008, 8.16)	0.08 (0.02, 5.56)	0.003
Interleukin-6 (pg/mL)	3.65 (0.97, 83.0)	3.91 (0.28, 78.7)	3.64 (0.5, 30.1)	3.45 (0.84, 37.6)	3.82 (0.97, 83.0)	0.75

Q1: lowest quartile, Q2: middle-low quartile, Q3: middle-high quartile, Q4: highest quartile.

*P value for differences of the variables among oxidized HDL tertiles.

^†^Adjusted for albumin. HDL: high-density lipoprotein, LDL: low-density lipoprotein, apoA-I: apolipoprotein A-I, apoA-II: apolipoprotein A-II, apoA-II/apoA-I: ratio of apoB to apoA-I, apoB: apolipoprotein B, apoB/apoA-I: ratio of apoB to apoA-I, hs-CRP: high-sensitivity CRP

### Associations of apoA-I and apoA-II with all-cause and CVD-related mortality and with composite CVD events

During follow-up, 78 patients (19%) died, of whom 28 patients died due to CVD-related diseases, and 112 composite CVD events occurred.

Quartiles of apolipoproteins and oxidized HDL were not associated with all-cause mortality (Table 3). However, significant associations were seen with composite CVD events (Table 4). Quartiles of apoA-I were not associated with composite CVD events (Table 4). Quartiles of apoA-II values were associated with decreased risk of composite CVD events in models adjusted for age, sex, dialysis vintage, DM, history of CVD and malnutrition (Table 4, Model 1). However, this association disappeared when adjusted using hs-CRP (Table 4, Model 2). Quartiles of apoB/apoA-I and oxidized HDL were independently associated with CVD events in models adjusted with and without hs-CRP (Table 4, Models 1 and 2). Moreover, these associations were confirmed in models adjusted with each together and confounders including hs-CRP or IL-6 (Table 4, Models 4 and 6).

**Table 3:** Cox hazard models of quartiles for apolipoproteins and oxidized HDL for all-cause mortality.

	Model 1	Model 2	Model 3	Model 4	Model 5	Model 6
ApoA-I Q1	Ref.	Ref.	-	-	-	-
Q2	1.03 (0.53, 2.00)	1.06 (0.55, 2.00)	-	-	-	-
Q3	0.93 (0.45, 1.94)	1.19 (0.60, 2.34)	-	-	-	-
Q4	0.73 (0.22, 2.28)	0.82 (0.33, 2.00)	-	-	-	-
ApoA-II Q1	Ref	Ref.	-	Ref.	Ref.	Ref.
Q2	0.99 (0.53, 1.48)	0.86 (0.45, 1.59)	-	0.82 (0.88, 1.99)	0.88 (0.47, 1.61)	1.03 (0.55, 1.95)
Q3	0.53 (0.24, 1.03)	**0.45 (0.20, 0.93)**	-	**0.42 (0.20, 0.82)**	**0.48 (0.23, 0.95)**	0.58 (0.27, 1.19)
Q4	1.00 (0.40, 2.07)	0.85 (0.38, 1.84)	-	0.70 (0.41, 1.73)	0.77 (0.51, 1.47)	0.97 (0.48, 1.29)
ApoA-II/apoA-I Q1	Ref.	-	Ref.	-	-	-
Q2	0.94 (0.48, 1.79)	-	0.89 (0.45, 1.76)	-	-	-
Q3	0.78 (0.38, 1.62)	-	0.66 (0.32, 1.40)	-	-	-
Q4	0.84 (0.40, 1.76)	-	0.75 (0.35, 1.60)	-	-	-
ApoB Q1	Ref.	Ref.	Ref.	-	-	-
Q2	1.37 (0.53, 2.19)	1.12 (0.59, 2.14)	1.28 (0.62, 2.62)	-	-	-
Q3	1.02 (0.43, 2.43)	0.87 (0.43, 1.73)	1.51 (0.64, 3.57)	-	-	-
Q4	0.75 (0.27, 2.08)	0.67 (0.31, 1.44)	1.17 (0.40, 3.37)	-	-	-
ApoB/apoA-I Q1	Ref.	-	-	Ref.	Ref.	Ref.
Q2	1.38 (0.67, 3.29)	-	-	0.98 (0.51, 2.02)	1.05 (0.54, 2.09)	1.08 (0.56, 2.14)
Q3	1.23 (0.50, 3.06)	-	-	0.74 (0.35, 1.49)	0.71 (0.35, 1.46)	0.65 (0.31, 1.35)
Q4	1.19 (0.43, 2.89)	-	-	0.54 (0.24, 1.07)	0.57 (0.27, 1.19)	0.52 (0.25, 1.11)
Oxidized HDL Q1	Ref.	Ref.	Ref.	Ref.	Ref.	Ref.
Q2	0.70 (0.73, 1.29)	0.71 (0.38, 1.30)	0.69 (0.37, 1.27)	0.73 (0.39, 1.35)	0.74 (0.40, 1.36)	0.71 (0.38, 1.31)
Q3	0.76 (0.40. 1.45)	0.86 (0.40. 1.64)	0.75 (0.39, 1.42)	0.87 (0.45, 1.66)	0.92 (1.47, 1.76)	0.90 (0.46, 1.72)
Q4	0.51 (0.25, 1.05)	0.57 (0.38, 1.15)	**0.47 (0.22, 0.95)**	0.56 (0.27, 1.11)	0.58 (0.28, 1.15)	0.53 (0.26, 1.06)

Q1: the lowest quartile, Q2: middle-low quartile, Q3: middle-high quartile, Q4: the highest quartile. Model 1: Each independent quintiles of apolipoprotein (apo) A-I, apoA-II, apoA-I/apo-II, apoB, apoB /apoA1 or oxidized high density lipoprotein (HDL) adjusted for age (years), sex (male vs. female), hemodialysis vintage (months), diabetes mellitus (yes vs. no), history of cardiovascular disease (yes vs. no), malnutrition (yes vs. no), HDL-cholesterol (C) and low density lipoprotein (LDL)-C. Model 2: Quintiles of apoA-I, apoA-II, apoB and oxidized HDL adjusted with confounders in model 1. Model 3: Quintiles of apoA-I/apoA-II, apoB and oxidized HDL adjusted with confounders in model 1. Model 3: Quintiles of apoA-II, apoB/apoA-I and oxidized HDL adjusted with confounders in model 1. Model 4: Quintiles of apoA-II, apoB/apoA-I and oxidized HDL adjusted with log high-sensitivity CRP. Model 5: Quintiles of apoA-II, apoB/apoA-I and oxidized HDL adjusted with log interleukin (IL) IL-6.

**Table 4:** Cox hazard models of quartiles for apolipoproteins and oxidized HDL for composite cardiovascular disease events.

	Model 1	Model 2	Model 3	Model 4	Model 5	Model 6
ApoA-I Q1	Ref.	Ref.	Ref.	-	Ref.	-
Q2	0.97 (0.56, 1.67)	1.04 (0.58, 1.86)	1.42 (0.81, 2.50)	-	1.64 (0.94, 2.87)	-
Q3	0.64 (0.34, 1.19)	0.74 (0.38, 1.44)	0.91 (0.43, 1.48)	-	1.26 (0.67, 2.34)	-
Q4	0.39 (0.14, 1.02)	0.48 (0.17, 1.29)	0.65 (0.28, 1.49)	-	0.72 (0.31, 1.61)	-
ApoA-II Q1	Ref.	Ref.	Ref.	Ref.	Ref.	Ref.
Q2	**0.55 (0.32, 0.84)**	**0.53 (0.30, 0.92)**	0.61 (0.34, 1.04)	**0.57 (0.33, 0.97)**	0.69 (0.39, 1.20)	0.64 (0.38, 1.12)
Q3	**0.44 (0.26, 0.73)**	**0.50 (0.27, 0.91)**	**0.50 (0.27, 0.90)**	**0.47 (0.27, 0.82)**	0.58 (0.31, 1.07)	**0.57 (0.32, 0.99)**
Q4	**0.58 (0.33, 0.98)**	0.65 (0.32, 1.26)	0.80 (0.3.9, 1.62)	0.72 (0.40, 1.26)	0.98 (0.46, 2.04)	0.89 (0.48, 1.61)
ApoA-II/apoA-I Q1	Ref.	Ref.	-	-	-	-
Q2	0.89 (0.51, 1.52)	0.92 (0.51, 1.66)	-	-	-	-
Q3	0.54 (0.29, 1.00)	0.51 (0.25, 1.01)	-	-	-	-
Q4	0.92 (0.51, 2.94)	0.99 (0.53, 1.84)	-	-	-	-
ApoB Q1	Ref.	Ref.	Ref.	-	Ref.	-
Q2	0.99 (0.58, 1.68)	1.11 (0.64, 1.91)	1.06 (0.61. 1.82)	-	1.00 (0.58. 1.71)	-
Q3	1.36 (0.75, 2.53)	2.06 (0.98, 4.39)	1.75 (0.91, 3.40)	-	1.77 (0.93, 3.44)	-
Q4	1.23 (0.66, 2.28)	**2.38 (1.03, 4.49)**	1.51 (0.77, 3.01)	-	1.53 (0.77, 3.06)	-
ApoB/apoA-I Q1	Ref.	Ref.	-	Ref.	-	Ref.
Q2	0.94 (0.60, 1.51)	1.31 (0.76, 2.26)	-	0.98 (0.59, 1.66)	-	0.92 (0.55, 1.55)
Q3	1.20 (0.73, 2.03)	**2.37 (1.17, 4.80)**	-	1.43 (0.81, 2.57)	-	1.28 (0.73, 2.30)
Q4	**1.83 (1.00, 3.57)**	**5.48 (2.09, 16.49)**	-	**2.21 (1.13, 4.56)**	-	**2.12 (1.09, 4.33)**
Oxidized HDL Q1	Ref.	Ref.	Ref.	Ref.	Ref.	Ref.
Q2	1.38 (0.85, 2.23)	1.41 (0.84, 2.39)	1.46 (0.86, 2.47)	1.46 (0.87, 2.48)	1.41 (0.84, 2.40)	1.40 (0.83, 2.38)
Q3	**2.19 (1.31. 3.75)**	**2.23 (1.29. 3.46)**	**2.20 (1.25, 3.39)**	**2.13 (1.23, 3.83)**	**2.14 (1.16, 3.18)**	**1.99 (1.15, 3.57)**
Q4	**2.00 (1.20, 3.38)**	**2.19 (1.26, 3.88)**	**2.19 (1.25, 3.91)**	**2.25 (1.30, 3.99)**	**2.04 (1.17, 3.62)**	**2.16 (1.24, 3.82)**

Q1: the lowest quartile, Q2: middle-low quartile, Q3: middle-high quartile, Q4: the highest quartile. Model 1: Each independent quartile of apolipoprotein (apo) A-I, apoA-II, apoA-I/apoA-II, apoB, apoB/apoA-I or oxidized high density lipoprotein (HDL) adjusted for age (years), sex (male vs. female), hemodialysis vintage (months), diabetes mellitus (yes vs. no), history of cardiovascular disease (yes vs. no), malnutrition (yes vs. no), HDL-cholesterol (C) (mg/dL), and low density lipoprotein (LDL)-C (mg/dL). Model 2: Each independent quartile of apoA-I, apoA-II, apoA-I/apoA-II, apoB, apoB/apoA-I or oxidized HDL adjusted for body mass index (kg/ m^2^), albumin (g/dL), log high-sensitivity (hs) CRP, statin use (yes vs. no) and confounders in model 1. Model 3: Quartiles of apoA-I, apoA-II, apoB and oxidized HDL adjusted with log oxidized LDL, log hs-CRP and confounders in model 1. Model 4: Quartiles of apoA-II, apoB/apoA-I and oxidized HDL adjusted with log oxidized LDL, log hs-CRP and confounders in model 1. Model 5: Quartiles of apoA-I, apoA-II, apoB and oxidized HDL adjusted with log oxidized LDL, log interleukin (IL) IL-6 and confounders in model 1. Model 6: Quartiles of apoA-II, apoB/apoA-I and oxidized HDL adjusted with log oxidized LDL, log IL-6 and confounders in model 1.

Associations of apolipoproteins, apoB/apoA-I and oxidized HDL with composite CVD events were also estimated in Cox hazard models of a 1-SD increase-of-variables (Table 5). Oxidized HDL, apoA-I and apoB, but not apoA-II, were independent risk factors in models adjusted for confounders including hs-CRP (Table 5, Model 3). However, the association of apoB disappeared in models adjusted with IL-6 instead of hs-CRP (Table 5, Model 5). Independent associations of apoB/apoA-I as well as oxidized HDL with CVD events were confirmed in models adjusted for confounders including hs-CRP (Table 5, Model 4). However, these associations disappeared in models adjusted for IL-6 instead of hs-CRP (Table 5, Model 6).

**Table 5:** Cox hazard models of events associated with composite cardiovascular disease events.

Per 1-SD increase	Model 1	Model 2	Model 3	Model 4	Model 5	Model 6
ApoA-I	**0.65 (0.47, 0.90)**	**0.67 (0.43, 3.41)**	**0.62 (0.43, 0.90)**	-	**0.69 (0.48, 0.99)**	-
ApoA-II	0.85 (0.62, 1.07)	0.91 (0.71, 1.16)	0.97 (0.74, 1.23)	0.93 (0.66, 1.29)	1.04 (0.79, 1.35)	0.99 (0.78, 1.27)
ApoA-II/apoA-I	1.04 (0.85, 1.28)	1.07 (0.87, 1.32)	-	-	-	-
ApoB	1.25 (0.92, 1.69)	1.27 (0.92, 1.76)	**1.40 (1.00, 193)**	-	1.38 (0.99, 1.89)	-
ApoB/apoA-I	**1.42 (1.09, 1.87)**	**1.39 (1.03, 1.88)**	-	**1.38 (1.04, 1.85)**	-	1.28 (0.96, 1.70)
Oxidized HDL	**1.45 (1.16, 1.83)**	**1.49 (1.28, 3.10)**	**1.44 (1.13, 1.81)**	**1.41 (1.12, 1.79)**	**1.37 (1.07, 1.73)**	**1.35 (1.05, 1.69)**

Model 1: Each independent variable adjusted with age (years), sex (male vs. female), hemodialysis vintage (months), diabetes mellitus (yes vs. no), history of cardiovascular disease (yes vs. no), malnutrition (yes vs. no), high-density lipoprotein (HDL)-cholesterol (C), low density lipoprotein (LDL)-C. Model 2: Each independent variable adjusted with body mass index (kg/ m^2^), albumin (g/dL), log high-sensitivity (hs)-CRP, statin use (yes vs. no) and confounders in model 1. Model 3: apolipoprotein (apo) A-I, apoA-II, apoB and oxidized HDL were adjusted with log oxidized LDL, log hs-CRP and confounders in model 1. Model 4: ApoA-II, apoB/apoA-I, oxidized HDL were adjusted with log oxidized LDL, log hs-CRP and confounders in model 1. Model 5: ApoA-I, apoA-II, apoB and oxidized HDL were adjusted with log oxidized LDL, log interleukin (IL)-6, and confounders in model 1. Model 6: ApoA-II, apoB/apoA-I, oxidized HDL were adjusted with log oxidized LDL, log IL-6 and confounders in model 1.

Associations between quartiles of oxidized HDL-to-apoA-I ratio (oxidized HDL/apoA-I) or 1-SD increment of oxidized HDL/apoA-I and all-cause mortality and CVD events were assessed to correct for the influence of the quantity of apoA-I to oxidized HDL (S2 Table). However, the findings in models using oxidized HDL/apoA-I resembled those in models using oxidized HDL alone (S2 Table). Oxidized HDL/apoA-I was not associated with all-cause mortality in multivariate Cox hazard models. However, oxidized HDL/apoA-I, but not apoA-II, was independently associated with composite CVD events.

Cox proportional hazard models for composite CVD events adjusted for time-varying covariates of HDL-C and hs-CRP are shown S3 Table. Each variable of oxidized HDL and apoB/apoA-I was an independent biomarker of composite CVD events in those models adjusted for the time-varying covariates of HDL-C and hs-CRP (S3 Table, Models 1 and 4). Independent associations of both factors with composite CVD events were confirmed in models adjusted with each together (S3 Table, Models 2 and 6), but the association of apoB/apoA-I with composite CVD events disappeared when adjusted for IL-6 (S3 Table, Model 3).

## Discussion

The present study showed that oxidized HDL could be a risk factor, rather than apoA-I, apoA-II, apoA-II/apoA-I or apoB, for CVD events among patients on prevalent HD. These findings resemble those of our previous study, which assessed associations among HDL-C, oxidized HDL, IL-6, and composite CVD events [17]. However, associations of apoB/apoA-I with CVD events resembled those of oxidized HDL with CVD events.

*Post hoc* analysis of the 4D study independently associated high apoA-II levels with decreased risk of all-cause mortality [8]. The present study showed that a high concentration of apoA-II did not decrease the risks for all-cause or composite CVD events in case-mix models or in a model adjusted with oxidized HDL and inflammatory biomarkers, or in a model adjusted with oxidized HDL/apoA-I and inflammatory biomarkers. ApoA-II thus may not be associated with all-cause mortality and CVD events compared with oxidized HDL and IL-6.

Compared with apoA-II, the highest quartile of apoB/apoA-I was independently associated with CVD events in Cox proportional hazard models adjusted with hs-CRP or IL-6 and time-varying Cox proportional hazard models with hs-CRP. These associations were confirmed in the models of values per 1-SD increment with adjustment for hs-CRP, and oxidized HDL was independently associated with CVD events in these models. These findings were the same as previously reported [9]. However, the association of apoB/apoA-I with CVD events disappeared with adjustment for IL-6 instead of hs-CRP. Discrepancies in associations of apoB/apoA-I with CVD events in the different models may be associated with a non-linear association of apoB/apoA-I with CVD events and such associations in the highest quartile of apoB/apoA-I may be gradually diminished when adjusted with IL-6. CRP production could be regulated by IL-6 stimulation, in which case IL-6 might exert a greater effect on clinical outcomes than CRP [21, 26]. In the present study, apoA-I was inversely associated with inflammatory biomarkers and positively associated with oxidized HDL, whereas apoB did not correlate with hs-CRP, IL-6 or oxidized HDL. The balance among the increased catabolism of apoA-I under conditions of inflammation and oxidative stress [19, 20, 27], diminished catabolism of apoB compared to production [19], and increased production of oxidized HDL might thus be associated with CVD events in patients receiving HD.

Oxidized HDL and oxidized HDL/apoA-I were not associated with all-cause mortality in the present study. Biomarkers of inflammation were independently associated with all-cause mortality. Oxidized HDL was estimated by ELISA using anti-oxidized apoA-I antibody, so data from this ELISA were dependent on the quantity of oxidized apoA-I. Our previous study associated causes of death with oxidized HDL levels determined using the same method; levels of oxidized HDL in patients who died due to non-CVD-related diseases were lower than in those who died due to CVD-related diseases [17]. Malnourished patients under HD displayed high levels of oxidized HDL [24], but the amount of oxidized HDL seems to be decreased in patients on HD with concomitant inflammation [18]. Oxidized HDL was lower in patients with DM that may be associated with inflammation. Patients with DM showed greater levels of inflammation than those without DM in the present study. ApoA-I particles are catabolized and modified by inflammation and oxidative stress, as mentioned above [19, 20, 27], resulting in decreased quantities of apoA-I. Lowered apoA-I would then be associated with lower concentrations of oxidized HDL. The unique metabolism of apoA-I in patients with HD who have comorbidities might thus influence circulating levels of apoA-I and oxidized HDL. Consequently, apoA-I might not be associated with survival in patients on HD [8], and the association of oxidized HDL with non-CVD-related mortality might also be decreased in patients on HD. In this regard, which of apoB/apoA-I, apoA-II or oxidized HDL represented the better biomarker for all-cause mortality remained unclear, since we could not identify significant associations between apolipoproteins and all-cause mortality.

Oxidized HDL is not only a biomarker for CVD events, but also potentially associated with the pathogenesis of atherosclerosis. Oxidized HDL and dysfunctional apoA-I modified by oxidation accumulate in atherosclerotic plaque [28, 29]. Moreover, oxidative stress modifies paraoxonase 1 (PON1), which binds to HDL with atheroprotective effects through the prevention of LDL oxidation [30]. The activity of PON1 is decreased in patients with CKD [31, 32]. Patients on HD with increased oxidized HDL might therefore show not only excessively modified HDL and apoA-I, but also dysfunctional PON1 caused by oxidation that results in vascular disease. Further investigations are needed to assess the associations between dysfunctional PON1 and CVD events in patients under HD.

High apoA-II/apoA-I and HDL particles containing both apoA-I and apoA-II were associated with increased risk of CVD events in population with low HDL-C and high CRP levels [33]. These conditions were common in patients under HD, confirming an association between apoA-II/apoA-I, oxidized HDL and CVD events. However, no significant relationships were evident between apoA-II/apoA-I and CVD events in this study.

The present findings must be considered with the following caveats. The patient cohort was relatively small, and measurements of oxidized HDL, apolipoproteins (apoA-I, apoA-II and apoB) and IL-6 at a single point do not imply accurate evaluation of these biomarkers over time. In time-varying models, HDL-C and hs-CRP were measured as covariates, but other important variables such as IL-6 were not estimated as time-varying covariates. In the present study, IL-6 and hs-CRP were adjusted as confounders for inflammatory biomarkers. Our previous study confirmed the abilities of myeloperoxidase (MPO) and soluble intercellular adhesion molecule (ICAM)-1 in predicting CVD events as well as IL-6 and hs-CRP. However, MPO and soluble ICAM-1 did not affect the associations of oxidized HDL and IL-6 with CVD events [17]. A recent study showed associations of inflammatory biomarkers (hs-CRP, IL-6, tumor necrosis factor, soluble ICAM-1, soluble vascular cellular adhesion molecule 1) with CVD events and all-cause mortality in an incident HD population [34]; in that study, IL-6 was the only predictor of both CVD events and all-cause mortality in the study population [34]. IL-6 and hs-CRP were thus selected as confounders in the present study, although other inflammatory biomarkers are potentially associated with clinical outcomes. Smokers were accepted in the present study. Smoking causes accumulation of unsaturated aldehydes such as acrolein in the body, which alter HDL to dysfunctional HDL [35]. The findings from this study may thus have been influenced by other modifications. In the study, oxidized HDL was detected by ELISA using anti-oxidized monoclonal apoA-I antibody. This monoclonal antibody was produced by immunization with H_2_O_2_-oxidized apoA-I [25]. We also estimated ELISA using another anti-oxidized monoclonal apoA-I antibody in the same population, in which monoclonal antibody was produced by immunization with chloramine T oxidized apoA-I. However, the detection ability of oxidized HDL by ELISA using anti-chloramine T oxidized monoclonal apoA-I antibody was quite low. We therefore decided to use ELISA with anti-H_2_O_2_ oxidized monoclonal apoA-I antibody. However, oxidants such as isoprostanes and 4-hydroxynonenal may affect apoA-I modifications in patients under HD [36]. We will therefore need to estimate other anti-oxidized apoA-I antibodies immunized with oxidized apoA-I produced by other oxidants. This study did not confirm associations among oxidized HDL, lipoprotein(a) or a low molecular weight (LMW) isoform of apoA and all-cause mortality and CVD events, while a recent *post hoc* analysis of the 4D study showed that lipoprotein(a) and LMW apoA isoform were associated with all-cause mortality [37]. Moreover, we did not estimate the functions of HDL and oxidized HDL, although patients with end-stage kidney disease show concomitant dysfunctional HDL [15, 16, 38]. A large prospective study with more accurate quantitative assessment of dysfunctional HDL over time to atherogenesis and time-varying measurement of apolipoproteins is thus required to clarify the reliability of oxidized HDL values and apoB/apoA-I as risk factors for CVD events among patients on prevalent HD.

## Conclusion

Oxidized HDL seems more important as a risk factor for CVD events and CVD-related mortality than apoA-I or apoA-II in patients on prevalent HD. ApoB/apoA-I may be associated with CVD events in a similar manner to oxidized HDL, but those associations may be influenced by IL-6.

## Supporting information

S1 Table Spearman’s rank correlation analysis among biomarkers at baseline. (DOCX)

S2 Table Cox hazard models of the ratio of oxidized HDL to apoA1 for all-cause mortality and composite cardiovascular disease events. (DOCX)

S3 Table Cox hazard models of events associated with cardiovascular disease; time-varying covariate, HDL-C (tv HDL-C) and hs-CRP (tv hs-CRP). (DOCX)
