# Supplementary material for: Associations among apolipoproteins, oxidized high-density lipoprotein and cardiovascular events in patients on hemodialysis
Source: PLoS One. 2017 May 18;12(5):e0177980. doi: 10.1371/journal.pone.0177980 (PMC5436869; doi:10.1371/journal.pone.0177980)
Supplement: S1 Table — (DOCX) [file pone.0177980.s001.docx]

S1 Table. Spearman’s rank correlation analysis among biomarkers at baseline

|  | LDL-C | ApoA-I | ApoA-II | ApoA-II/apoA-I | ApoB | ApoB/apoA-I | Hs-CRP | Interleukin-6 | Oxidized HDL | Oxidized LDL |
| --- | --- | --- | --- | --- | --- | --- | --- | --- | --- | --- |
| HDL-C | 0.004 | 0.75^‡^ | 0.40^‡^ | -0.45^‡^ | -0.21^‡^ | -0.65^‡^ | -0.29^‡^ | -0.26^‡^ | 0.35^‡^ | -0.25^‡^ |
| LDL-C | - | 0.03 | 0.27^‡^ | 0.24^‡^ | 0.71^‡^ | 0.57^‡^ | 0.003 | -0.07 | -0.06 | 0.25^‡^ |
| ApoA-I |  | - | 0.59^‡^ | -0.42^‡^ | -0.09 | -0.64^‡^ | -0.27^‡^ | -0.23^‡^ | 0.30^‡^ | -0.02 |
| ApoA-II |  |  |  | 0.39^‡^ | 0.23^‡^ | -0.20^‡^ | -0.20^†^ | -0.21^‡^ | 0.08 | 0.09 |
| ApoA-II/apoA-I |  |  |  |  | 0.31^‡^ | -.48^‡^ | 0.14^†^ | 0.02 | -0.27^‡^ | 0.16^†^ |
| ApoB |  |  |  |  | - | 0.78^‡^ | 0.09 | 0.07 | -0.08 | 0.38^‡^ |
| ApoB/apoA-I |  |  |  |  |  | - | 0.26^‡^ | 0.22^‡^ | -0.24^‡^ | 0.36^‡^ |
| Hs-CRP |  |  |  |  |  |  | - | 0.52^‡^ | -0.12^*^ | 0.12^*^ |
| Interleukin-6 |  |  |  |  |  |  |  | - | -0.001 | 0.18^†^ |
| Oxidized HDL |  |  |  |  |  |  |  |  | - | -0.03 |

*: p<0.05, †: p<0.001, ‡: p<0.0001. HDL-C: high-density lipoprotein cholesterol, LDL-C: low-density lipoprotein cholesterol, apoA-I: apolipoprotein A-I, apoA-II: apolipoprotein A-II, apoA-II/apoA-I: ratio of apoA-II to apoA-I, apoB: apolipoprotein B, apoB/apoA1: ratio of apoB to apoA1, Hs-CRP: high sensitive CRP
