# Supplementary material for: Associations among apolipoproteins, oxidized high-density lipoprotein and cardiovascular events in patients on hemodialysis
Source: PLoS One. 2017 May 18;12(5):e0177980. doi: 10.1371/journal.pone.0177980 (PMC5436869; doi:10.1371/journal.pone.0177980)
Supplement: S2 Table — (DOCX) [file pone.0177980.s002.docx]

S2 Table. Cox hazard models of the ratio of oxidized HDL to apoA1 for all-cause mortality and composite cardiovascular disease events

|  | All-cause mortality | | | Cardiovascular events | |
| --- | --- | --- | --- | --- | --- |
| Quintiles | Model 1 | Model 2 | Model 3 | | Model 4 |
| ApoA-II Q1 | Ref | Ref | Ref | | Ref |
| Q2 | 1.09 (0.56, 2.11) | 1.23 (0.62, 2.44) | 0.61 (0.34, 1.06) | | 0.72 (0.40, 1.28) |
| Q3 | 0.63 (0.29, 1.32) | 0.75 (0.33, 1.65) | **0.50 (0.28, 0.91)** | | 0.61 (0.32, 1.13) |
| Q4 | 1.44 (0.62, 3.25) | 1.78 (0.75, 4.18) | 0.67 (0.34, 1.31) | | 0.85 (0.41, 1.59) |
| ApoB Q1 | Ref | Ref | Ref | | Ref |
| Q2 | 1.54 (0.75, 3.18) | 1.50 (0.73, 3.11) | 1.18 (0.60, 2.13) | | 1.11 (0.56, 2.17) |
| Q3 | 1.71 (0.70, 4.21) | 1.57 (0.63, 3.83) | **2.68 (1.27, 5.74)** | | **2.56 (1.20, 5.49)** |
| Q4 | 1.44 (0.46, 4.31) | 1.42 (0.46, 4.21) | **3.31 (1.33, 8.91)** | | **3.02 (1.22, 7.44)** |
| Oxidized HDL/apoA-I 　 Q1 | Ref | Ref | Ref | | Ref |
| Q2 | 1.11 (0.55, 2.22) | 1.11 (0.55, 2.22) | 0.82 (0.40, 1.64) | | 0.83 (0.41, 1.64) |
| Q3 | 0.99 (0.49, 1.97) | 0.99 (0.49, 1.97) | **1.84 (1.02, 3.34)** | | **1.87 (1.05, 3.41)** |
| Q4 | 0.69 (0.35, 1.39) | 0.69 (0.35, 1.39) | **2.05 (1.16, 3.73)** | | **1.91 (1.08, 3.47)** |
| Per 1-SD increase | Model 5 | Model 6 | Model 7 | | Model 8 |
| ApoA-II | 0.93 (0.69, 1.27) | 1.00 (0.72, 1.38) | 0.90 (0.70, 1.16) | | 1.00 (0.78, 1.29) |
| ApoB | 1.26 (0.83, 1.86) | 1.25 (0.83, 1.85) | 1.39 (0.99, 1.94) | | 1.39 (0.99, 1.94) |
| Oxidized HDL/apoA-I | 0.88 (0.68, 1.13) | 0.88 (0.69, 1.12) | **1.42 (1.17, 1.73)** | | **1.38 (1.14, 1.69)** |

Models 1, 3: Quartiles of oxidized HDL/apoA-I ratio was adjusted with quartiles of apoA-II and apoB, age (years), sex (male vs. female), hemodialysis vintage (months), diabetes mellitus (yes vs. no), history of cardiovascular disease (yes vs. no), malnutrition (yes vs. no), HDL-cholesterol (C) and low density lipoprotein (LDL)-C (mg/dL), log oxidized LDL and log hsCRP.

Models 2, 4: Quartiles of oxidized HDL/apoA-I ratio were adjusted with quartiles of apoA-II and apoB, age, sex, hemodialysis vintage, diabetes mellitus, history of cardiovascular disease, malnutrition, HDL-C and LDL-C, log oxidized LDL and log interleukin (IL)-6.

Models 5, 7: Oxidized HDL/apoA-I ratio per 1-SD increment was adjusted with per 1-SD increments in apoA-II and apoB, age, sex, hemodialysis vintage, diabetes mellitus, history of cardiovascular disease, malnutrition, HDL-C and LDL-C, log oxidized LDL and log hsCRP.

Models 6, 8: Oxidized HDL/apoA-I ratio per 1-SD increment was adjusted with per 1-SD increments in apoA-II and apoB, age, sex, hemodialysis vintage, diabetes mellitus, history of cardiovascular disease, malnutrition, HDL-C and LDL-C, log oxidized LDL and log IL-6.
