# Supplementary material for: Associations among apolipoproteins, oxidized high-density lipoprotein and cardiovascular events in patients on hemodialysis
Source: PLoS One. 2017 May 18;12(5):e0177980. doi: 10.1371/journal.pone.0177980 (PMC5436869; doi:10.1371/journal.pone.0177980)
Supplement: S3 Table — (DOCX) [file pone.0177980.s003.docx]

S3 Table. Cox hazard models of events associated with cardiovascular disease; time-varying covariate, HDL-C (tv HDL-C) and hs-CRP (tv hs-CRP)

|  | tv HDL-C (n = 369) | | | tv hs-CRP (n = 369) | | | |
| --- | --- | --- | --- | --- | --- | --- | --- |
|  | Model 1 | Model 2 | Model 3 | | Model 4 | Model 5 | Model 6 |
| ApoA-I | 0.98 (0.98, 1.00) | - | - | | **0.99 (0.97, 0.99)** | 0.98 (0.98, 1.00) | - |
| ApoA-II | 0.97 (0.92, 1.03) | 0.99 (0.94, 1.05) | 1.01 (0.96, 1.06) | | 0.97 (0.92, 1.02) | 0.98 (0.92, 1.05) | 0.97 (0.91, 1.02) |
| ApoB | **1.01 (1.00, 1.03)** | - | - | | **1.07 (1.00, 1.03)** | **1.02 (1.00,1.04)** | - |
| ApoB/ApoA1 | **3.89 (1.32, 11.48)** | **4.37 (1.41, 13.55)** | 3.06 (0.93, 10.07) | | **5.80 (1.62, 20.86)** | - | **5.52 (1.50, 20.29)** |
| Log oxidized HDL | **2.20 (1.50, 3.25)** | **2.18 (1.48, 3.22)** | **2.06 (1.34, 3.04)** | | **2.16 (1.45, 3.21)** | **2.07 (1.38, 3.11)** | **2.05 (1.37, 3.09)** |

Model 1: tv high density lipoprotein (HDL)-cholesterol (C) adjusted with each independent variable of apolipoprotein (apo) A-I, apoA-II, apoB, apoB/apoA-I or oxidized HDL and confounders of age (years), sex (male vs. female), hemodialysis vintage (months), diabetes mellitus (yes vs. no), malnutrition (yes vs. no), and low density lipoprotein (LDL)-C (mg/dL, C).

Model 2: tv HDL-C adjusted with apoA-II, apoB/apoA-I, log oxidized HDL and confounders in model 1.

Model 3: tv HDL-C adjusted with apoA-II, apoB/apoA-I, log oxidized HDL, log interleukin-6 and confounders in model 1.

Model 4: tv high sensitive (hs)-CRP adjusted with independent variable of apoA-I, apoA-II, apoB, apoB/apoA-I or oxidized HDL, and confounders in model 1 and baseline HDL-C.

Model 5: tv hs-CRP adjusted with apoA-I, apoA-II, apoB, log oxidized HDL, HDL-C and confounders in model 1.

Model 6: tv hs-CRP adjusted with apoA-II, apoB/apoA-I, log oxidized HDL, HDL-C and confounders in model 1.

Bold means statistical significant findings.
